# Proceedings of the Local Dental Association

**Published:** 1860-11

**Authors:** T. F. Davenport

**Affiliations:** Cincinnati


### Proceedings of Societies.

---

CINCINNATI, TUESDAY EVE., Oct. 9, 1860.

Local Dental Association met pursuant to adjournment at Dr. Davenport's office. Members present:—Drs. H. A. Smith, Taft, Richardson, Cameron, James, Foot and Davenport.

Minutes of previous meeting read and approved.

Dr. L. A. Hendrich was elected to membership.

The regular topic for discussion, viz: "*Toothache,*" was taken up.

Dr. TAFT: There are several varieties of this affection—an exposed nerve becomes inflamed, and from the same causes which operate in other soft parts, there will be congestion, and consequent pressure upon the nerve fibrils, producing pain, which will be confined to the tooth where it originates. In neuralgic toothache the pain follows the ramifications of the nerves. The former, or inflammatory variety, would be relieved by depletion, which would be of little or no avail in neuralgia. Pain from inflammation of a nerve is of a throbbing character, dull, heavy and continuous—in neuralgia it is of a more lancinating character, and may affect other teeth sympathetically at the same time—

stated a case of a lower molar tooth with nerve exposed—all the teeth around to the bicuspid on the opposite side aching, this was of neuralgic character.

Dr. RICHARDSON spoke of Sympathetic Toothache. Pain is often felt in a sound tooth, have noticed it more particularly in connection with the lower wisdom teeth—the upper molars of the same side will often be affected. This variety of Toothache may be caused by the inflammation from a diseased tooth passing along the nerve to the main branch and there the pain may be reflected to other *sound teeth*, receiving their filaments of nerve from the same branch. Do not recognize neuralgia without inflammation.

Dr. TAFT: Neuralgic pain may be accompanied with inflammation. Neuralgic toothache, often becoming chronic, can not, as a rule, be relieved except by the extraction of the tooth where the difficulty originates; have saved some cases but failed in most. Inflammatory toothache is more easily cured. In the case of a patient of a nervous temperament, having severe toothache—extending to many teeth, would, if possible, extract the tooth. Have many times during the past four months, depleted inflamed pulps, covered the point of exposure with cotton saturated with creosote, and filled over with gold,—have had no trouble with them yet, can not tell whether it will be permanent or not.

Dr. H. A. SMITH thinks neuralgic toothache can exist without inflammation or exposure of the nerve. Filled a tooth over a slightly exposed nerve, but was obliged to extract the tooth afterward. Would not attempt to cure acute inflammatory toothache; thinks the chronic variety more manageable.

Dr. RICHARDSON: Neuralgia is an affection of the nerve itself. Mentioned a class of cases which he sometimes meets. Deep-seated decay, tooth bone softened to the nerve, but not decomposed, has in his practice left the layer of softened dentine over the nerve and filled without any pain; in a short time the tooth will begin to ache—thinks pulp was diseased before the operation, and had been relieving itself by serous exudation, through the softened dentine. The filling interfered with that process, hence the pain.

Dr. Foote: Succeeds in saving most of the aching teeth which are presented. Abjures all medicinal agents; has not used creosote for four months.

Dr. Richardson inquired if plaster of Paris would not answer for temporary fillings.

Dr. Foote (continued). Saves teeth where there is a discharge of matter, by *mopping* out the root and filling the fang to the apex, at one sitting.

Dr. Taft: When a tooth is discharging through the root, the matter is secreted by the parts around the apex—if the root be filled, and the abscess remains, the matter will still be secreted and must find some other way of escape.

Inflammatory toothache can often be relieved by counter-irritation. Creosote excites the absorbents to increased action; and also forms an insoluble compound with the serum, which acts as a protection to the nerve.

Dr. Foote: Thinks when a root has an abscess, the sac will produce sufficient irritation to excite the absorbents.

Dr. Hendrich was appointed Essayist for next meeting; subject for discussion—"How soon after extraction of natural teeth should artificial dentures be inserted.'

Adjourned to meet at the office of Dr. Cameron, on the second Tuesday in November, 1860.

T. F. DAVENPORT, *Sec'y.*

